# The gamma-glutamyl transpeptidase to platelet ratio: a good noninvasive biomarker for predicting for the occurrence and prognosis of patients with hepatitis E virus-related acute liver failure

**DOI:** 10.3389/fmed.2025.1573114

**Published:** 2025-03-24

**Authors:** Shuai Zhu, Jun Yuan, Feng Ju, Mengmeng Gu, Ze Xiang, Yan Zhang

**Affiliations:** ^1^Department of Clinical Laboratory, The Fifth People’s Hospital of Suzhou/The Affiliated Infectious Diseases Hospital, Suzhou Medical College of Soochow University, Suzhou, China; ^2^Department of Laboratory Medicine, The First People’s Hospital of Yancheng City, Yancheng, China; ^3^Department of Laboratory Medicine, The Yangzhou University Jianhu Clinical College, Jianhu, China; ^4^Department of Clinical Laboratory, The Affiliated Suzhou Hospital of Nanjing Medical University, Suzhou Municipal Hospital, Gusu School, Nanjing Medical University, Suzhou, China; ^5^Zhejiang University School of Medicine, Hangzhou, China; ^6^Department of Clinical Laboratory, Suzhou Yongding Hospital, Suzhou, China

**Keywords:** hepatitis E, acute liver failure, gamma-glutamyl transpeptidase to platelet ratio (GPR), diagnosis, prognosis

## Abstract

**Background:**

The relationship between GPR and hepatitis E has not been reported. This study evaluates the GPR levels in AHE patients and HEV-ALF patients and explores the role of GPR levels in the prognosis of HEV-ALF patients, offering new strategies and methods for the diagnosis and prognosis of HEV-ALF patients.

**Methods:**

Serum samples were collected from 206 AHE patients and 217 HEV-ALF patients admitted to the First Affiliated Hospital of Zhejiang University School of Medicine, Suzhou Yongding Hospital, and Nanjing Medical University Affiliated Suzhou Hospital between January 1, 2017, and November 30, 2024.

**Results:**

The GPR level in the HEV-ALF group was significantly higher than in the AHE group (*p* < 0.001). OPLS-DA analysis revealed that INR, TBIL, PLT, GPR, TCH, ALT, AFP, TP, ALB, and AST were the major influential factors for the occurrence of HEV-ALF. GPR showed good diagnostic performance with an AUC of 0.701. DCA further supported the utility of GPR across a range of threshold probabilities. Regarding the prediction of 30-day mortality, GPR levels were significantly higher in the non-survival group compared to the survival group (*p* < 0.001). OPLS-DA analysis highlighted GPR as the most influential factor for predicting 30-day mortality. GPR demonstrated an AUC of 0.703. DCA results also indicated GPR’s strong decision-making ability for predicting 30-day mortality in HEV-ALF patients.

**Conclusion:**

GPR is highly expressed in HEV-ALF patients and is closely related to their prognosis.

## Introduction

Hepatitis E is a liver disease caused by the hepatitis E virus (HEV) and is one of the main causes of acute viral hepatitis (AVH) worldwide (1, 2). HEV is primarily transmitted through the fecal-oral route (3). Currently, there are four well-characterized genotypes of HEV that infect mammals (4). Genotypes 1 and 2 are restricted to humans, while genotypes 3 and 4 cause zoonotic infections with a wide range of hosts (5, 6). Although hepatitis E typically results in asymptomatic infection and self-limiting disease, some patients exhibit typical symptoms of acute hepatitis and may even develop acute liver failure (ALF), leading to death, particularly in pregnant women, the elderly, and those with underlying liver disease. The global mortality rate for HEV is 1–2%, but in developing countries, about 20–40% of acute hepatitis E patients may progress to acute liver failure. This rate increases to 50% in pregnant women with HEV infection and up to 75% in patients with pre-existing liver conditions (7, 8). Therefore, timely diagnosis of HEV-related acute liver failure (HEV-ALF) is crucial.

The treatment of acute liver failure emphasizes early diagnosis and treatment, active prevention of complications, and dynamic assessment of the patient’s condition. Accurate and efficient early prognosis evaluation is essential for preventing the deterioration of acute liver failure (9, 10). Currently, TBIL, prothrombin or INR, serum creatinine, prealbumin, and platelet count have some value in assessing the prognosis of acute liver failure (11, 12). However, these indicators cannot precisely evaluate the patient’s prognosis, and there are no specific prognostic biomarkers for HEV-ALF in clinical practice (13). Therefore, identifying effective new prognostic biomarkers for HEV-ALF patients is beneficial for precise diagnosis and can effectively prevent the progression of liver disease, holding significant clinical importance.

In recent years, a new simple laboratory index, the gamma-glutamyl transpeptidase (GGT)-to-platelet ratio (GPR), has been primarily used as a clinical indicator for diagnosing fibrosis (14, 15). Gamma-glutamyl transpeptidase (GGT) plays an important physiological role, especially in the absorption, distribution, and synthesis of amino acids and proteins (16). In normal individuals, serum GGT mainly originates from the liver. Therefore, abnormalities in this indicator may suggest liver dysfunction, such as chronic hepatitis, liver cirrhosis, intrahepatic bile duct inflammation, alcoholic liver disease, and liver cancer (17, 18). In patients with liver cirrhosis, portal hypertension can lead to splenomegaly and hypersplenism, which inhibit bone marrow function and result in decreased platelet count (16). Thus, when both GGT and platelet count are abnormal, the GPR value changes (19). Through biochemical testing, it is possible to further determine whether liver function is abnormal, providing good diagnostic significance and value for viral hepatitis such as hepatitis B or liver cirrhosis. However, the relationship between GPR and hepatitis E has not been reported. This study evaluates the GPR levels in AHE patients and HEV-ALF patients and explores the role of GPR levels in the prognosis of HEV-ALF patients, offering new strategies and methods for the diagnosis and prognosis of HEV-ALF patients.

## Patients and methods

### Study populations

In this study, serum samples were collected from 206 AHE patients and 217 HEV-ALF patients admitted to the First Affiliated Hospital of Zhejiang University School of Medicine, Suzhou Yongding Hospital, and Nanjing Medical University Affiliated Suzhou Hospital between January 1, 2017, and November 30, 2024. Clinical baseline characteristics, laboratory indicators, length of stay, and diagnostic data were recorded at admission and discharge. Patients were followed up for 30 days post-discharge. Survival data were collected through medical records or direct contact with patients or their families, with death or liver transplantation as the study endpoints. This study was recorded in the National Human Resources Database (CJ1253) and approved by the Ethics Committee of the First Affiliated Hospital of Zhejiang University School of Medicine (2020454) and the Ethics Committee of Suzhou Hospital Affiliated to Nanjing Medical University (K2022080H01). All subjects signed informed consent forms.

The diagnostic criteria for hepatitis E are based on the “Diagnostic Criteria for Viral Hepatitis E (WS-2008)” issued by the Ministry of Health in 2008 (20). The specific criteria are as follows: (1) positive serum anti-HEV IgM, and/or a more than two-fold increase in anti-HEV IgG titer, and/or detectable HEV RNA. (2) Clinical manifestations consistent with acute hepatitis, including elevated liver enzymes and/or jaundice and/or non-specific symptoms such as fatigue, itching, and nausea.

The diagnostic criteria for hepatitis E-related acute liver failure (HE-ALF) are based on the “Guidelines for Diagnosis and Treatment of Liver Failure (2012 edition)” (21). The specific criteria are as follows: Acute onset, with hepatic encephalopathy (HE) of grade 1 or higher occurring within 2 weeks, accompanied by the following manifestations: (1) extreme fatigue, along with significant anorexia, abdominal distension, nausea, vomiting, and other severe gastrointestinal symptoms. (2) Progressive deepening jaundice in the short term, with serum total bilirubin ≥10 times the upper limit of normal or daily increase ≥17.1 μmol/L. (3) Bleeding tendency, with prothrombin activity ≤40% or international normalized ratio (INR) ≥1.5, excluding other causes. (4) Progressive liver shrinkage.

The exclusion criteria for patients were: (1) co-infection with other hepatitis viruses. (2) Receipt of drugs that may cause severe liver damage within 3 months before admission. (3) Presence of malignant diseases, including hepatocellular carcinoma. (4) Blood disorders. (5) Pregnancy. (6) Prior liver transplantation. (7) Co-infection with HIV or parasites. (8) Autoimmune diseases.

### Anti-HEV IGM and IGG antibodies detection

To detect anti-HEV IgM and IgG antibodies, HEV enzyme-linked immunosorbent assay (ELISA) kits (WANTAI BioPharm, Beijing, China) were employed. The assay determined positivity based on the optical density (OD) in serum samples. An OD *>*1.1 was designated as positive, while an OD ≤1.1 was deemed negative.

### HEV RNA detection

To detect HEV RNA, real-time quantitative PCR (qPCR) was employed. The extraction of total viral RNA from serum samples was carried out using Aikang virus nucleic acid purification kits (Aikang MedTech Co., Ltd., Shenzhen, China).

### GPR calculation

GPR scores were calculated as described previously using the following formula [where the upper limit of normal (ULN) for GGT = 24 U/L] (22):


$$GPR=\frac{GGT\;\left[U/L\right]/\mathrm{upper limit of normal}\;\left(ULN\right)\;}{\mathrm{Platelet count}\;\left[10^{9}/L\right]}\times 100$$

### Statistical analysis

SPSS 22.0 and GraphPad Prism 9 were used for statistical analysis. The measurement data of normal distribution were expressed as mean ± standard deviation ( $\bar{x\;}$ ± s), and a *t*-test was used for inter-group comparison. The measurement data of non-normal distribution were expressed as median (quartile), and Mann–Whitney *U* test was used for comparison between groups. The chi-square test was used to compare the data groups. SIMCA was utilized for data analysis, employing orthogonal partial least squares discriminant analysis (OPLS-DA). The MedCalc software (MedCalc Software Ltd., Ostend, Belgium) was employed to calculate the area under the receiver operating characteristic curve (AUC), sensitivity, and specificity. To assess the utility of GPR for assisted decision-making at various threshold probabilities, decision curve analysis (DCA) was used. A *p* < 0.05 was considered statistically different.

## Results

### Baseline characteristics of the enrolled patients

There were no significant differences in age and sex between the AHE and HEV-ALF groups (both *p* > 0.05). Statistically significant differences were observed in the ALT, AST, total protein, albumin, TBIL, INR, total cholesterol, alpha-fetoprotein, and platelet count between the two groups (*p* < 0.05). In contrast, there were no statistically significant differences in the levels of white blood cell count, C-reactive protein, gamma-glutamyltransferase, urea nitrogen, and creatinine (*p* > 0.05) (Table 1). Notably, the GPR level in the HEV-ALF group was significantly higher than that in the AHE group (*p* < 0.001).

**Table 1:** Analysis of baseline characteristics of AHE and HEV-ALF population.

Variables	AHE group (*n* = 206)	HEV-ALF group (*n* = 217)	*p*
Age (years)	57.03 ± 13.58	58.16 ± 11.56	0.362
Gender (male/female)	119/87	138/79	0.220
WBC (10^9^/L)	5.83 (4.58, 7.58)	6.30 (4.86, 7.80)	0.094
ALT (U/L)	531.00 (227.75, 931.50)	701.00 (281.95, 1501.00)	0.003
AST (U/L)	239.00 (116.75, 624.50)	440.50 (123.50, 852.70)	0.009
GGT (U/L)	144.00 (76.88, 230.25)	149.90 (101.60, 228.45)	0.070
TP (g/L)	63.14 ± 6.58	60.27 ± 7.55	<0.001
ALB (g/L)	36.35 ± 5.67	34.23 ± 5.18	<0.001
TBIL (μmol/L)	38.82 (24.66, 115.00)	173.69 (97.02, 262.90)	<0.001
UREA (mmol/L)	5.08 (3.69, 6.46)	4.61 (3.52, 6.84)	0.447
CR (μmol/L)	69.00 (54.78, 101.62)	67.00 (54.20, 97.75)	0.183
INR	1.07 (1.00, 1.25)	1.84 (1.63, 2.10)	<0.001
CRP (mg/L)	9.68 (3.84, 21.78)	12.50 (5.80, 19.85)	0.154
TCH (mmol/L)	3.84 ± 1.24	3.22 ± 1.14	<0.001
AFP (ng/mL)	41.30 (3.80, 161.08)	78.90 (7.66, 319.25)	0.003
PLT (10^9^/L)	206.94 ± 76.99	150.28 ± 65.17	<0.001
GPR	0.84 (0.47, 1.15)	1.18 (0.78, 1.57)	<0.001

### Diagnostic performances for the occurrence of HEV-ALF patients

**Figure 1:**
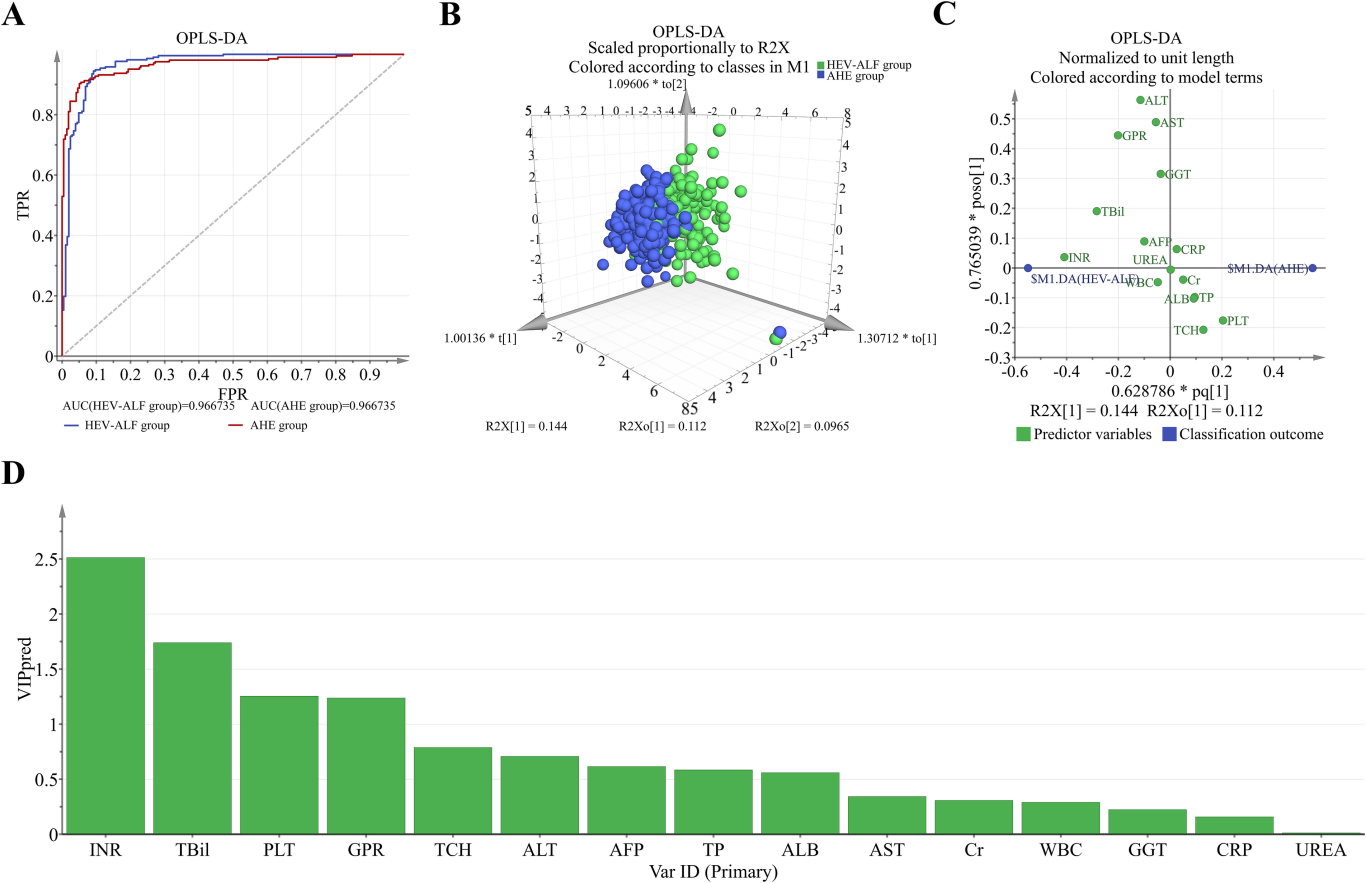
OPLS-DA was used to evaluate the diagnostic performances for the occurrence of HEV-ALF patients. **(A)** Receiver operating characteristics between the HEV-ALF and AHE patients. **(B)** The HEV-ALF and AHE patients can be well distinguished. **(C)** The relation of each parameter to the predictive component and first orthogonal component was revealed. **(D)** The more left, the higher predictive VIP pred value.

OPLS-DA analysis was performed to evaluate and rank the influences of the baseline parameters on the occurrence of HEV-ALF patients. Distinct dot clusters of the AHE group and the HEV-ALF group are observed in Figures 1A,B. Loading plot revealed 10 parameters as major influential factors for the occurrence of HEV-ALF patients (i.e., INR, TBIL, PLT, GPR, TCH, ALT, AFP, TP, ALB and AST level) (Figures 1C,D).

**Figure 2:**
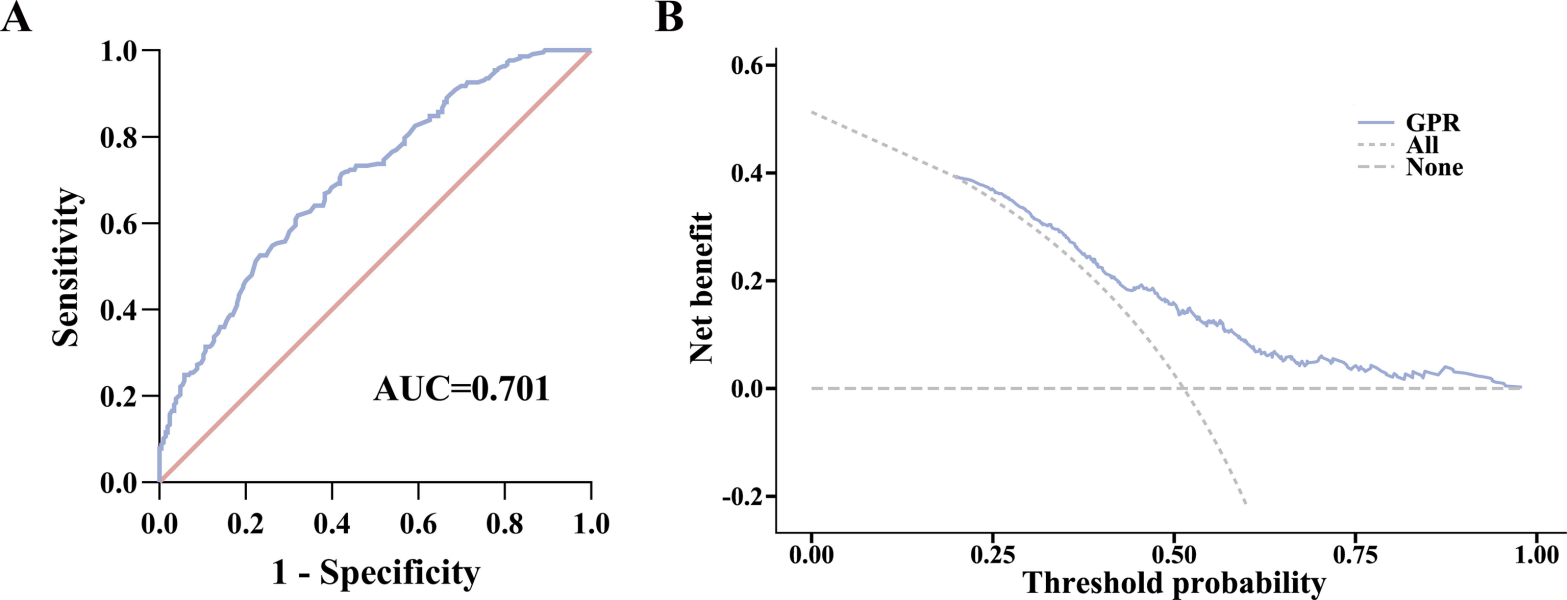
Prediction ability of GPR for HEV-ALF occurrence. **(A)** ROC curve analysis. **(B)** Evaluation of the benefit in assisting decision-making at different threshold probabilities using DCA.

GPR demonstrated good diagnostic utility to detect the occurrence of HEV-ALF patients with an AUC of 0.701 [95% confidence interval (CI) (0.655–0.745); *p* < 0.0001] (Figure 2A). A cut-off GPR value of 1.04 demonstrated optimal sensitivity of 61.75% and specificity of 67.96% with a positive predictive value (PPV) of 67.0% and negative predictive value (NPV) of 62.8%.

In addition, decision curve analysis (DCA) was employed to further evaluate the net benefit of GPR in assisting decision-making at different threshold probabilities. As shown in Figure 2B, GPR demonstrated consistent performance across the entire threshold probability range.

### Prediction capability in the 30-day mortality of patients with HEV-ALF

A total of 217 HEV-ALF patients were followed up for 30 days, and 36 of them died. Therefore, we were divided into 30-day HEV-ALF survival group and HEV-ALF death group. Baseline analysis revealed that the GPR level in the HEV-ALF non-survival group was significantly higher than that in the HEV-ALF survival group (*p* < 0.001) (Table 2).

**Table 2:** Analysis of baseline characteristics of HEV-ALF survival group and HEV-ALF death group.

Variables	HEV-ALF survival group (*n* = 181)	HEV-ALF death group (*n* = 36)	*p*
Age (years)	57.65 ± 11.90	60.72 ± 9.38	0.145
Gender (male/female)	114/67	24/12	0.675
WBC (10^9^/L)	6.30 (5.08, 7.90)	6.20 (4.52, 7.45)	0.334
ALT (U/L)	683.80 (243.25, 1559.00)	869.60 (442.42, 1365.20)	0.370
AST (U/L)	440.50 (123.50, 895.25)	444.95 (106.20, 793.00)	0.664
GGT (U/L)	148.70 (96.95, 224.50)	170.65 (117.50, 259.12)	0.116
TP (g/L)	60.67 ± 7.58	58.26 ± 7.15	0.081
ALB (g/L)	34.44 ± 5.34	33.16 ± 4.23	0.178
TBIL (μmol/L)	148.80 (78.97, 247.71)	309.31 (178.20, 358.35)	<0.001
UREA (mmol/L)	4.51 (3.52, 6.72)	5.36 (3.44, 7.42)	0.224
CR (μmol/L)	66.40 (54.65, 93.50)	68.70 (45.38, 103.62)	0.963
INR	1.79 (1.60, 2.07)	2.06 (1.82, 2.66)	<0.001
CRP (mg/L)	12.41 (5.60, 19.85)	13.65 (6.89, 20.36)	0.600
TCH (mmol/L)	3.21 ± 1.18	3.26 ± 0.98	0.819
AFP (ng/mL)	77.64 (9.23, 314.34)	79.50 (5.63, 388.81)	0.838
PLT (10^9^/L)	153.72 ± 66.22	132.97 ± 57.35	0.081
GPR	1.14 (0.76, 1.45)	1.70 (1.06, 2.49)	<0.001

**Figure 3:**
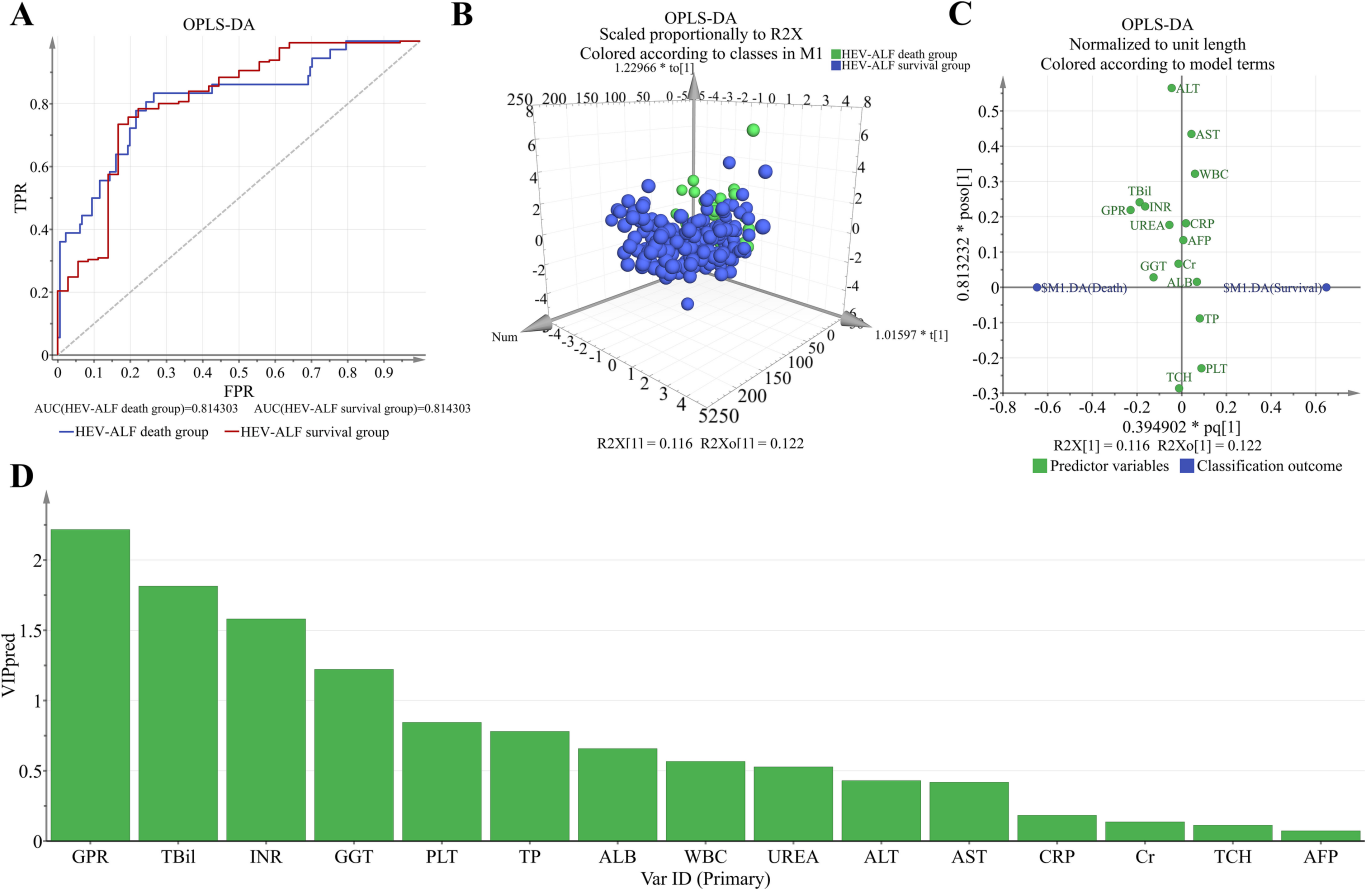
OPLS-DA was used to evaluate and rank the influences of the baseline parameters on the 30-day mortality of HEV-ALF patients. **(A)** Receiver operating characteristics between the HEV-ALF survival group and death group. **(B)** The HEV-ALF survival group and death group can be well distinguished. **(C)** The relation of each parameter to the predictive component and first orthogonal component was revealed. **(D)** The more left, the higher predictive VIP pred value.

OPLS-DA analysis was performed to evaluate and rank the influences of the baseline parameters on the 30-day mortality of HEV-ALF patients. Distinct dot clusters of the HEV-ALF survival group and the HEV-ALF death group are observed in Figures 3A,B. Loading plot revealed GPR as the first influential factor for prediction capability in the 30-day mortality of HEV-ALF patients (Figures 3C,D).

**Figure 4:**
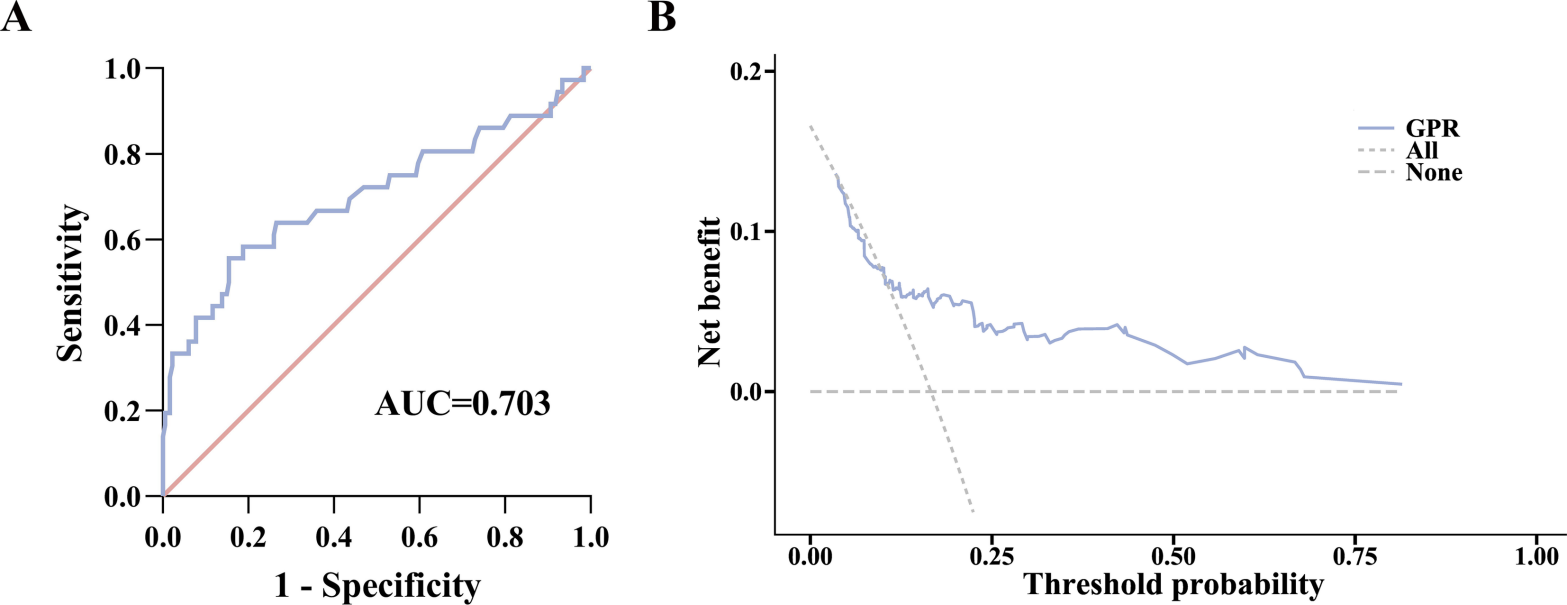
Prediction ability of GPR for the 30-day mortality of patients with HEV-ALF. **(A)** ROC curve analysis. **(B)** Evaluation of the benefit in assisting decision-making at different threshold probabilities using DCA.

The AUC of prediction capability of GPR in the 30-day mortality of patients with HEV-ALF was 0.703 (0.637–0.763), with sensitivity of 55.56% and specificity of 84.53% (Figure 4A). In addition, DCA findings also indicated that GPR exhibited high decision-making ability for predicting the 30-day mortality (Figure 4B).

## Discussion

The mortality rate of acute hepatitis E infection is very low, possibly less than 1%, but the prognosis is worse in elderly patients and those with chronic liver disease. Severe vertical transmission of acute hepatitis E infection in pregnant women can lead to acute liver failure in more than half of the newborns (23). In the process of hepatitis E virus (HEV) infection leading to acute liver failure, immunosuppressed patients may experience persistent hepatitis symptoms and viremia, and the infection can transition from acute to chronic (24). Although the overall prognosis of HEV-related liver failure is relatively good, the mortality rate can exceed 50% in patients who cannot receive emergency liver transplantation (25). Accurate early diagnosis and prognosis assessment can help clinicians develop the best treatment plan, improve the clinical efficacy of HEV-ALF, and reduce patient mortality.

The γ-glutamyltransferase (GGT) to platelet/(PLT) ratio (GPR) is a commonly used indicator for assessing liver function, reflecting the progression of liver lesions, especially during the hyperactive synthesis or bile excretion obstruction phase within the liver during the process of fibrotic tissue proliferation and repair (26). Serum GGT levels can show a significant increase during these phases. In the progression from chronic hepatitis B (CHB) to cirrhosis, the intrahepatic portal venous pressure gradually increases, which can lead to splenic congestion and enlargement, resulting in hypersplenism and a decrease in platelet count, thereby causing an elevation in the GGT/PLT ratio. However, the accuracy of using blood indicators to evaluate liver parenchymal lesions is relatively poor (17). In this study, we first compared the GPR levels between the AHE group and the HEV-ALF group, and the results showed that the GPR level in the HEV-ALF group was significantly higher than that in the AHE group. OPLS-DA analysis also indicated that GPR is an important factor affecting the progression to severe disease in AHE patients. The ROC level for GPR in predicting the progression to severe disease in AHE patients reached 0.701, and decision curve analysis (DCA) showed that GPR performed well across the entire range of threshold probabilities. As a non-invasive and clinically common detection indicator, GPR has significant clinical value and is highly beneficial for clinical practice, especially for widespread application and promotion in primary healthcare institutions.

More significantly, GPR demonstrated superior performance in assessing the prognosis of HEV-ALF patients. Among the 217 HEV-ALF patients divided into 30-day survival and mortality groups, the GPR level was notably higher in the mortality group compared to the survival group. OPLS-DA analysis found that loading plot revealed GPR as the first influential factor for prediction capability in the 30-day mortality of HEV-ALF patients. Subsequent evaluation of GPR’s predictive capacity for 30-day mortality in HEV-ALF patients showed an area under the curve (AUC) of 0.703 (0.637–0.763), with a sensitivity of 55.56% and specificity of 84.53%, indicating that GPR serves as a promising novel biomarker for prognostic assessment in HEV-ALF patients.

This study has the following limitations: first, it lacks data on the dynamic changes in GPR levels, and the correlation between GPR levels and the progression of HEV-ALF patients’ conditions warrants further investigation. Second, although the study population is from multiple centers, the genotypes of the study population were not analyzed, and the value of GPR in different genotype populations was not assessed. Third, it lacks a comparison of diagnostic efficacy between GPR and conventional indicators such as INR and MELD in this study. Fourth, the mechanism by which GPR mediates the progression of severe hepatitis E is still unclear.

In summary, this study shows that GPR is highly expressed in HEV-ALF patients and is closely related to their prognosis. GPR has the potential to become a good new non-invasive marker for assessing the severity and prognosis of hepatitis E patients.

## Data Availability

The original contributions presented in the study are included in the article/supplementary material, further inquiries can be directed to the corresponding author.

## References

[1] Liu T Cao Y Weng J Gao S Jin Z Zhang Y. Hepatitis E virus infects human testicular tissue and Sertoli cells. Emerg Microbes Infect. (2024) 13:2332657. doi: 10.1080/22221751.2024.2332657, PMID: 38517709 PMC11057402

[2] Xiang Z He X-L Zhu C-W Yang J-J Huang L Jiang C. Animal models of hepatitis E infection: advances and challenges. Hepatobiliary Pancreat Dis Int. (2024) 23:171–80. doi: 10.1016/j.hbpd.2023.10.001, PMID: 37852916

[3] Wu J Wang H Xiang Z Jiang C Xu Y Zhai G. Role of viral hepatitis in pregnancy and its triggering mechanism. J Transl Int Med. (2024) 12:344–54. doi: 10.2478/jtim-2024-0015, PMID: 39360164 PMC11444475

[4] Wu J Xiang Z Gao C Huang L Hua J Tong L. Genotype 4 HEV infection triggers the initiation and development of acute pancreatitis. Microbes Infect. (2023) 25:105190. doi: 10.1016/j.micinf.2023.105190, PMID: 37499789

[5] Jiao F Zhao Y Zhou G Meng C Wang L Wu S. Multiple functions of hepatitis E virus ORF3. Microorganisms. (2024) 12:1405. doi: 10.3390/microorganisms12071405, PMID: 39065173 PMC11278674

[6] You M Zheng F Zhou T Xu Y Wu J Zhuo S. Metabolic profiling and early diagnosis of alcoholic fatty liver disease using support vector machine model. Adv Gut Microbiome Res. (2024) 2024:5744974. doi: 10.1155/2024/5744974

[7] Brüggemann Y Klöhn M Wedemeyer H Steinmann E. Hepatitis E virus: from innate sensing to adaptive immune responses. Nat Rev Gastroenterol Hepatol. (2024) 21:710–25. doi: 10.1038/s41575-024-00950-z, PMID: 39039260

[8] Xiang Z Jiang C Yang J Huang L Jiang B Wang X. Serum extracellular vesicle-derived ASS1 is a promising predictor for the occurrence of HEV-ALF. J Med Virol. (2023) 95:e28425. doi: 10.1002/jmv.28425, PMID: 36562411

[9] Fernandez J Bassegoda O Toapanta D Bernal W. Acute liver failure: a practical update. JHEP Rep. (2024) 6:101131. doi: 10.1016/j.jhepr.2024.101131 39170946 PMC11337735

[10] Martínez-Martínez L Rosales-Sotomayor G Jasso-Baltazar E Torres-Díaz J Aguirre-Villarreal D de León IH-D. Acute liver failure: management update and prognosis. Rev Gastroenterol Mex. (2024) 89:404–17. doi: 10.1016/j.rgmxen.2024.05.002 39033039

[11] Nogueira AF Teixeira C Fernandes C Moinho R Gonçalves I Pinto CR. Prognostic markers in pediatric acute liver failure. GE Port J Gastroenterol. (2024) 31:165–72. doi: 10.1159/000531269, PMID: 38757064 PMC11095588

[12] Shen Y Xu W Chen Y Wen S Chen Q Liu S. Early prediction of acute-on-chronic liver failure development in patients with diverse chronic liver diseases. Sci Rep. (2024) 14:28245. doi: 10.1038/s41598-024-79486-w, PMID: 39548240 PMC11568263

[13] Wu J Guo N Zhang X Xiong C Liu J Xu Y. HEV-LFS: a novel scoring model for patients with hepatitis E virus-related liver failure. J Viral Hepat. (2019) 26:1334–43. doi: 10.1111/jvh.13174, PMID: 31294523

[14] Purkayastha S Jha AK Kumar R Dayal VM Jha SK. Serum gamma-glutamyl transpeptidase-to-platelet ratio as a noninvasive marker of liver fibrosis in chronic hepatitis B. Cureus. (2023) 15:e33744. doi: 10.7759/cureus.33744, PMID: 36793825 PMC9925026

[15] Shneider BL Goodrich NP Ye W Sawyers C Molleston JP Merion RM. Nonfasted liver stiffness correlates with liver disease parameters and portal hypertension in pediatric cholestatic liver disease. Hepatol Commun. (2020) 4:1694–707. doi: 10.1002/hep4.1574, PMID: 33163838 PMC7603532

[16] Luo Q-Q Li Q-N Cai D Jiang S Liu S-S Liu M-S. The index sAGP is valuable for distinguishing atypical hepatocellular carcinoma from atypical benign focal hepatic lesions. J Hepatocell Carcinoma. (2024) 11:317–25. doi: 10.2147/JHC.S443273, PMID: 38348099 PMC10860805

[17] Ghachem I Hamzaoui L Bachali A Rhimi C Medhioub M Mahmoudi M. Performance of GPR score for non-invasive assessment of liver fibrosis in chronic hepatitis B Tunisian patients. Tunis Med. (2024) 102:715–21. doi: 10.62438/tunismed.v102i10.5091, PMID: 39441156 PMC11574378

[18] Wang J Xia J Yan X Yang Y Wei J Xiong Y. The gamma-glutamyl transpeptidase to platelet ratio predicts liver inflammation in chronic hepatitis B with normal or mildly elevated alanine transaminase. Clin Res Hepatol Gastroenterol. (2020) 44:913–22. doi: 10.1016/j.clinre.2020.01.011 32147439

[19] Zhan J Wang J Zhang Z Xue R Jiang S Liu J. Noninvasive diagnosis of significant liver inflammation in patients with chronic hepatitis B in the indeterminate phase. Virulence. (2023) 14:2268497. doi: 10.1080/21505594.2023.2268497, PMID: 37938933 PMC10653690

[20] Wu J Shi C Sheng X Xu Y Zhang J Zhao X. Prognostic nomogram for patients with hepatitis E virus-related acute liver failure: a multicenter study in China. J Clin Transl Hepatol. (2021) 9:828–37. doi: 10.14218/JCTH.2020.00117, PMID: 34966646 PMC8666371

[21] Wu J Zhang X Liu H Guo N Pan Q Wang Y. RDW, NLR and RLR in predicting liver failure and prognosis in patients with hepatitis E virus infection. Clin Biochem. (2019) 63:24–31. doi: 10.1016/j.clinbiochem.2018.11.012, PMID: 30502317

[22] Calvopina DA Lewindon PJ Ramm LE Noble C Hartel GF Leung DH. Gamma-glutamyl transpeptidase-to-platelet ratio as a biomarker of liver disease and hepatic fibrosis severity in paediatric cystic fibrosis. J Cyst Fibros. (2022) 21:236–42. doi: 10.1016/j.jcf.2021.10.014, PMID: 34953741

[23] Wu C Wu X Xia J. Hepatitis E virus infection during pregnancy. Virol J. (2020) 17:73. doi: 10.1186/s12985-020-01343-9, PMID: 32522266 PMC7286216

[24] Alexandrova R Tsachev I Kirov P Abudalleh A Hristov H Zhivkova T. Hepatitis E virus (HEV) infection among immunocompromised individuals: a brief narrative review. Infect Drug Resist. (2024) 17:1021–40. doi: 10.2147/IDR.S449221, PMID: 38505248 PMC10948336

[25] Wolski A Pischke S Ozga A-K Addo MM Horvatits T. Higher risk of HEV transmission and exposure among blood donors in Europe and Asia in comparison to North America: a meta-analysis. Pathogens. (2023) 12:425. doi: 10.3390/pathogens12030425, PMID: 36986347 PMC10059948

[26] Narkewicz MR. Cystic fibrosis liver disease in the post-modulator era. Curr Opin Pulm Med. (2023) 29:621–5. doi: 10.1097/MCP.0000000000001017, PMID: 37678151

